# Is psychological science progressing? Explained variance in PsycINFO articles during the period 1956 to 2022

**DOI:** 10.3389/fpsyg.2022.1089089

**Published:** 2022-12-21

**Authors:** Geir Smedslund, Jan Ketil Arnulf, Jan Smedslund

**Affiliations:** ^1^Norwegian Institute of Public Health, Oslo, Norway; ^2^Diakonhjemmet Hospital, Oslo, Norway; ^3^BI Norwegian Business School, Oslo, Norway; ^4^Department of Psychology, University of Oslo, Oslo, Norway

**Keywords:** explained variance, semantics, scientific progress, context, empirical/*a priori*

## Abstract

We aimed to numerically assess the progress of modern psychological science. Average explained variance in 1565 included articles was 42.8 percent, and this was constant during 1956 to 2022. We explored whether this could be explained by a combination of methodological conventions with the semantic properties of the involved variables. Using latent semantic analysis (LSA) on a random sample of 50 studies from the 1,565, we were able to replicate the possible semantic factor structures of 205 constructs reported in the corresponding articles. We argue that the methodological conventions pertaining to factor structures will lock the possible explained variance within mathematical constraints that will make most statistics cluster around 40 percent explained variance. Hypotheses with close to 100 percent semantic truth value will never be part of any assumed empirical study. Nor will hypotheses approaching zero truth value. Hypotheses with around 40 percent truth value will probably be experienced as empirical and plausible and, consequently, as good candidates for psychological research. Therefore, to the extent that the findings were indeed produced by semantic structures, they could have been known without collecting data. Finally, we try to explain why psychology had to abandon an individual, causal method and switch to studying whether associations among variables at the group level differ from chance. Psychological processes take place in indefinitely complex and irreversibly changing contexts. The prevalent research paradigm seems bound to producing theoretical statements that explain each other to around 40%. Any theoretical progress would need to address and transcend this barrier.

## Introduction

An empirical science will continuously improve its ability to predict and explain its target phenomena and their inter-relations (Grayling, 2021). Modern scientific psychology is said to have started in 1879 when Wilhelm Wundt established the first experimental psychology lab. It seems that most psychologists take it for granted that psychology is continuously progressing. But is it true that psychology is increasing its ability to predict the strength of inter-relations among its variables? It also seems that most psychologists take it for granted that all associations among psychological variables are empirical. The distinctions between empirical/*a priori*, analytical/synthetical and contingent/non-contingent (Bradley and Swartz, 1979) are not taken into account in much psychological research, even though a number of writers have discussed these themes for a long time (Martin et al., 2015). We are concerned that many findings in psychology may be *pseudo-empirical* – i.e., understood as empirical and contingent when they actually are *a priori* and non-contingent (i.e., semantical) (Smedslund, 1991, 2016). To the extent that psychology is not an empirical science, one may wonder whether there can be any progress from an endless collection of data.

We wanted to numerically assess the progress that has been made in psychology’s ability to predict, and explain relations among psychological concepts. Much has been written about the state of psychology as a science since Cronbach and Meehl (see, e.g. (Cronbach, 1957; Meehl, 1978), but we are not aware of any attempt to *quantify* its progress over time. In section one of this paper, we report an attempt to do so, using explained variance as our metric. In section two, we explore a subset of the research from section one, using semantic algorithms to show and suggest how allegedly empirical associations can be replicated from semantical associations. We also propose that methodological conventions create a self-perpetuating loop of limited explained variance. Section three is a discussion based on sections one and two, in which we argue that more conceptual work, notably on the concept of context, is necessary to understand the existing limitations of psychology as a science. Solutions will not be easy, but we present an outline of what progress might look like. In the first two sections, we have separate methods and results sections. The third section is a general discussion.

## Empirical investigation of explained variance in psychology

### Materials and methods

In order to track psychology’s progress in explaining variance, we searched for articles in Ovid PsycINFO from inception until the end of 2021 (search date: April 26, 2022). PsycINFO is a bibliographic database of more than 5 million abstracts of literature in the field of psychology. It is produced by the American Psychological Association and distributed on the association’s APA PsycNET and through third-party vendors. It is the electronic version of the now discontinued Psychological Abstracts. PsycINFO provides records of journal articles from 1887. Our simple search strategy was: “explained variance” in Abstract OR “coefficient of determination” in Abstract OR “R-squared” in Abstract. Limits were “Human” and “English language”. If data on explained variance was reported in the abstract, we extracted this from the abstract. If not, we tried to obtain the full text and extracted the explained variance from this. If explained variance was reported for more than one group of variables in an article, we reported the largest explained variance. We excluded the following: articles in non-English languages, method papers, reviews, studies reported in Dissertation Abstracts International, and studies with no psychological variables (e.g. brain studies). For each year with at least one study with data on this, we counted the number of studies and computed an unweighted mean R-square with a 95 percent confidence interval. We also computed a grand mean over all years with a 95 percent confidence interval. The plots were created using the R package ‘ggplot2’ (Wickham, 2016). The raw data for the analyses are available on request from the corresponding author. This study was not preregistered. We did not receive research ethics committee (e.g., Institutional review board) approval as there were no participants involved.

## Results

The search resulted in 2480 records. We excluded 915 records. Table 1 shows the reasons for exclusion.

**TABLE 1:** Reasons for excluding articles (n).

Reason for exclusion	Number
Dissertations or conference abstracts	405
Could not obtain fulltext	182
Explained variance not reported and could not be estimated	131
Not psychology proper (brain science, etc.)	82
Methodological paper	60
Review article	29
Comment or editorial	15
Erratum or extracted	9
Non-English	1
Qualitative study	1
Total number excluded	915

**FIGURE 1:**
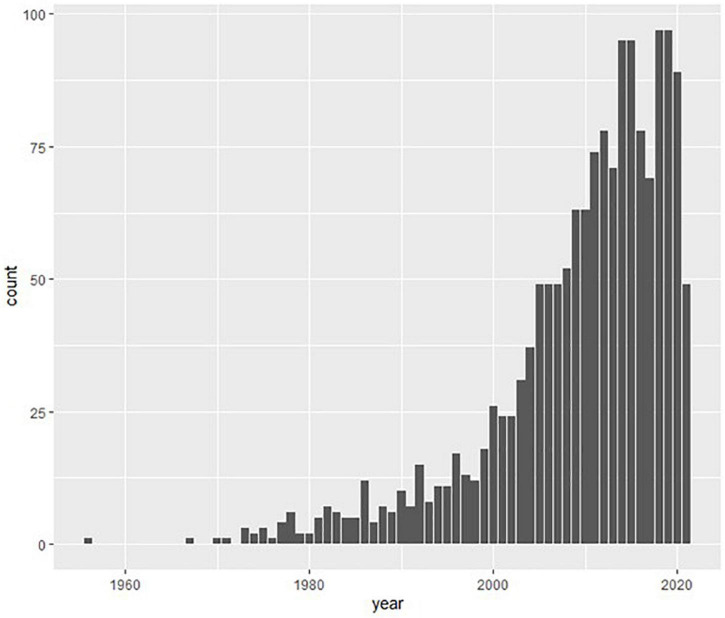
Number of publications per year with “explained variance,” coefficient of determination” or “R-squared” in the abstract in PsychINFO 1956-2021.

A total of 1,565 articles were included. The earliest was published in 1956. For all 1,565 articles, the mean amount of (maximum) explained variance was 42.8 percent with 95 percent confidence interval from 41.7 to 43.9. In other words, 57.2 percent of the variance in dependent variables in psychology was not explained by the researchers’ models and hypotheses. As shown in Figure 1, the number of publications mentioning explained variance has increased exponentially over the years (the seeming drop in the two most recent years is simply due to registers still being updated in the databases).

**FIGURE 2:**
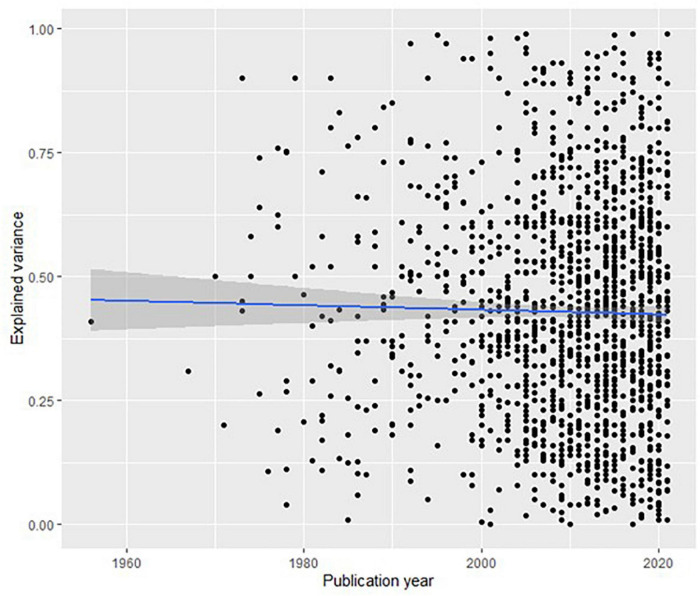
Dot plot of explained variance per year for 1,565 articles in PsycINFO (with 95% confidence intervals). Regression line models the mean explained variance per year.

The most important question is how much the explained variance has changed over time. Figure 2 shows that there has been virtually no change.

The regression coefficient of −0.0005 means that for every year the explained variance has decreased by 0.05 percent. In other words, the ability of scientific psychology to explain variance has not improved in 60 years of ever-increasing research activity.

## Discussion

Many readers may be struck by surprise or even disbelief by these findings. It is therefore important to ask whether the selection of articles reported here is representative for psychology as a whole. A listing of the 30 most frequent keywords in the included articles (Table 2) gives some indication.

**TABLE 2:** The 30 most frequent keywords in the included articles with frequencies of occurrence.

Keyword	Frequency	Keyword	Frequency
1. Test validity	158	16. Human sex differences	51
2. Psychometrics	134	17. Social support	49
3. Test reliability	97	18. Coping behavior	49
4. Test construction	91	19. Aging	49
5. Major depression	89	20. Rating scales	47
6. Quality of life	81	21. Models	46
7. Well being	77	22. Physical activity	42
8. Symptoms	74	23. Psychosocial factors	41
9. Personality traits	69	24. Anxiety	41
10. Stress	59	25. Questionnaires	40
11. Health	57	26. Demographic characteristics	40
12. Cognitive ability	57	27. Intention	39
13. Risk factors	56	28. College students	39
14. Self-efficacy	53	29. Measurement	38
15. Prediction	51	30. Age differences	37

The most frequent keywords are related to psychometrics, but among the top 30 keywords, there are terms from, e.g., abnormal psychology, positive psychology, personality psychology, stress, social psychology, and aging. And these are only the most frequent keywords. Our 1,565 included articles list a total of 2,939 unique keywords. It is hard to determine the exact meaning of “representativity” when referring to a complex field like psychology, and hence we have no uniform standard to judge from. However, we think that this sample is highly representative of any *search effort* to assess the power of today’s psychology in explaining phenomena under study. The surveyed database is the world’s most encompassing representation of psychological research containing more than 4 million bibliographic records (WoltersKluwer, 2022). By sampling all articles that meet our search criteria, we think that the criteria for representativity are met and that disputing this would require a different conception of explanatory power – a step that we would welcome in the debate.

Earlier articles tended to report only one or a few explained variances, whereas more recent articles did many more analyses, with some reporting as many as 20-30 different R-squares. In these cases we consistently reported the highest R-square in each article. In this way, we deliberately wanted to create a bias in favor of more recent articles. And, still, the explained variance has not increased. Based on arguments that are presented in sections two and three, we conducted a study to explore whether semantics could explain the findings of the first section.

## Using semantic algorithms to recreate findings from a subset of the studies

### Introduction

The purpose of this second study is to explore the possible reasons that could explain our two observations – first, that the average explained variance hovers around 42%, and second, that this number has been practically constant since 1956. We believe that the explanation may be found in the way that semantic relationships play out in the design of psychological studies and how these semantic relationships interact with our psychometric conventions.

Semantic relationships between psychological statements imply that the statements share meaning (Landauer, 2007). Such relationships will necessarily create correlations between the statements, since people who agree that one of the statements is true also tend to agree with the other statement. One example that actually appears in our dataset concerns two questions about marketing of fish: “How likely is it that you *expect* to eat fish for dinner” and “How likely is it that you *plan* to eat fish for dinner”. To the extent that someone *plans* to eat fish, they probably also *expect* to eat fish. It is therefore no surprise that two such statements correlate highly when data are sampled from human subjects. It is precisely this semantic or logical relationship that made Bertrand Russel argue that some scientific relationships can be constructed logically in absence of empirical data, a claim that later led to the evolution of latent “constructs” in psychology (Lovasz and Slaney, 2013). Semantic relationships are given *a priori*.

While Russell lamented on the cognitive challenge of agreeing on logical argumentation, even to trained logicians (Russell, 1922), we can now use digital algorithms as relatively impartial tools of measurement. Natural language processing (NLP) allows us to parse texts and calculate a quantitative measure of the degree to which the meaning of two statements overlaps (Landauer et al., 1998; Gefen et al., 2017). In Latent Semantic Analysis (LSA), a quantitative expression of meaning is defined as the degree to which two words or phrases may take each other’s place without loss of meaning (Landauer and Dumais, 1997). Applied to statements used as measurement items in a study, the technique can be used to estimate the degree to which their responses can be expected to overlap (Arnulf et al., 2018b).

In recent years, an expanding volume of research has demonstrated how semantic algorithms can predict and model data structures from survey research in psychology and related disciplines (e.g., Arnulf et al., 2014; Nimon et al., 2016; Gefen and Larsen, 2017; Kjell et al., 2019; Rosenbusch et al., 2020). The semantic algorithms can predict and replicate the correlation matrices of empirical studies (Arnulf et al., 2018c). This line of research raises a hitherto unsolved question about the relationship between semantic and empirical sources of information in published studies (Arnulf, 2020; Arnulf and Larsen, 2021). If the statistical relationships and models can be calculated prior to collecting data, the meaning and value of human responses becomes questionable, since it does not seem that our methods differ efficiently between empirical information and their *a priori* conceptual patterns (e.g., Lamiell, 2013; Maul, 2017; Slaney, 2017; Uher, 2021a).

The recently demonstrated pervasiveness of semantic structures in empirical datasets offers a possible reason for our observed relative rigidity in the explanatory capability of quantitative psychological research. Even though semantic relationships have only recently come under empirical scrutiny, they have in fact had a long history of tacit implications in the tradition of factor analysis, a major tool in psychological methods (Lamiell, 2013).

Since a large proportion of the studied variables in psychology are so-called “latent constructs”, they are usually measured by multiple single variables thought of as “indicators” of the latent construct (Borsboom, 2008). The nature of the indicators and their relationship to theory are usually established through statistical techniques such as factor analysis or principal component analysis, common tools of construct validation (Cronbach and Meehl, 1955; Bagozzi, 2011; Uher, 2021b). In this tradition, measurement items that are very similar (such as planning and expecting to eat fish) are automatically taken as indicators of the same latent variable. It is precisely the degree of semantic overlap that indicates their *a priori* relationships. To count as an indicator of a different variable, a measurement item must be recognizably different in meaning.

This has led to a controversy in psychometrics as to whether high semantic relationships are desirable or not (Kline, 2000): High semantic similarities within variables and difference between variables will give high measures of internal consistency such as Cronbach’s alpha and clear factor structures. However, one might argue logically that semantically similar measurements are redundant since they measure the same variable over again. Lower semantic similarities will create messier factor structures but might capture more “truly” empirical phenomena, i.e., variables that are connected in ways that do not have *a priori* relationships.

Without taking a position on this issue, we may describe the conventions of interpreting factor loadings in a way that will let us predict how semantics may influence the explained variance of quantitative studies. Let us assume the previously used example of measurement items related to eating fish, and proceed from very high to very low factor loadings:

1. Extremely high factor loadings (higher than about 0.90): One way of obtaining these would be to pose exactly the same question twice (“I plan to eat fish” - > “I plan to eat fish”). This would probably not happen in practice, as most people would intuitively see that the information from the second question is redundant. However, explaining one variable with itself would actually approach 100% explained variance.
2. Very high factor loadings (higher than about 0.60, but below the level of obvious tautologies): These are indicative of belonging to the same construct. Researchers want these because they create good statistics in the form of factor structures and alpha coefficients. For example, the two items “When eating fish for dinner at home I feel good” and “When eating fish for dinner at home I feel satisfied” have factor loadings of 0.82 and 0.81 on the same factor.
3. Medium high factor loadings (higher than about 0.40, but not high enough to indicate belonging to one single factor): These are usually difficult to interpret, and researchers will try to avoid them. The reason is that they tend to be interpreted as dubious representatives of the construct in focus, but even worse – they may load on other constructs as well, blurring the distinctions between variables. This can contribute to autocorrelations, common method variance and similar flaws. Therefore, medium high factor loadings tend to be omitted.
4. Low factor loadings (lower than 0.40): If a measurement item has a sufficiently high loading on one factor, it may still display lower loadings on the other factors, so-called cross-loadings. These are tolerated below thresholds that may vary from discipline to discipline, but generally below 0.40 or even 0.30. In our example, the item “I have been eating fish for a long time” loads 0.78 on its focus factor, but has a cross-loading of 0.47 on another factor and could therefore be suspected of causing trouble for the analysis.
5. True independence between variables would be indicated by high focus factor loadings and zero crossloadings but this is a very rare situation. This is most often the case where variables belong to “formative” constructs defined by theoretically argued but disjunct variables, such as eating fish and personal income where the relationship would be truly empirical with no *a priori* logical or semantic relations.

There is a mathematical consequence of these conventions that bears on explained variance and the possible semantic relationships in psychological studies. In a set of variables subjected to principal component analysis, the explained variance among the variables can be calculated from the factor structure alone. In fact, any factor will be explained by any other factor to the extent that there are cross-loadings. Specifically, when regressing factor A on factor B, the R^2^ will be equal to the cross-loadings of factor A on B, divided by the factor loadings within factor B.

Take again the fish-eating scale as example: The first factor (termed “satisfaction” with eating fish) measured by three items obtains (in our study) an average focus factor loading of 0.82. The orbiting factor termed “habit strength, also measured by three items, has a focus factor loading of 0.80. However, their average cross-loadings are 0.31. In this case, their mutual shared variance is equal to the cross-loadings (0.31) divided by the focus factor loadings (0.80 for factor B), which is 0.39. In other words, the cross-loadings on the factors will indicate an explained variance of 39% if someone should regress one factor on the other.

Going back to the interpretations of factor-loadings, it becomes apparent that high measures of explained variance will be rare, since factors are constructed to be separate. In other words, the high factor loadings indicative of high explained variances will be interpreted as belonging to the same latent construct. To count as a different variable, the factor analytical techniques require that the cross-loadings are substantially lower than the focus factor loadings. We argue that whatever the threshold a discipline sets, the ratio of crossloadings to focus factor loadings will always show a maximum around 40% of each other.

And this is precisely because the researchers will take semantics into account, as described above. Higher factor loadings on more than one factor will be interpreted as troublesome diffusion of meaning from one factor to another, disturbing the statistics. We simply do not want to calculate the explained variances of tautologies because they are redundant, and we also try to avoid measures that capture several variables at the same time. Therefore, our methodological conventions in factor analysis locks us at a maximum explained variance defined by the ratio of cross-loadings to focus factor loadings.

This is where the semantic properties of psychological measurements enter our methodological frameworks, defining the differences between theoretical and empirical analyses. From a purely semantic point of view, we do not want to explain one variable by itself (because it is a tautology). Also, if variables are semantically similar, we prefer to see them as facets of the same underlying (latent) variable. To avoid contaminating the relationships between variables, we demand that there is a gap between explanations within and between the variables. In plain words, this implies that these requirements should be detectable as semantic structures of studies in psychology *a priori* using semantic algorithms.

The present framework can also be used to explain and explore the difference between empirical and semantic relationships. Semantic relationships are predictable *a priori*. This can be seen as a Bayesian foundation for exploring the collected data (Krueger, 2001; Sprenger, 2009), where the semantic relationships make up the priors that we expect to meet. Empirical discoveries are the theoretically and significant departures from what is expected.

Based on the considerations above, we can argue that there will be a detectable semantic structure among most of the variables in a psychological study. The semantic structure may be explicitly argued through logically related concepts, or implicitly assumed as the independence between variables. In practice, we expect similar questions to elicit the same responses, and a prerequisite for eliciting different responses is to pose different questions.

We therefore have two related research questions for this second section: (1) Is it possible to detect a semantic structure in a random sample of the studies from section one, and (2) Will the average relationship between the semantically defined variables in these structures explain each other mutually at the predicted level of around 40% (confidence intervals including the 42.8% observed in section one)?

Note that we are only looking for the possibility that one semantic structure is discernible in the data, as detected by semantic algorithms. As shown in previous studies, certain subgroups of people seem to differ from each other in the fine details of how they interpret questionnaires. In such cases, demographics or other types of group characteristics can make groups deviate to some extent from what is semantically predicted (Arnulf et al., 2018a,^2020^). Also, the expected explained variance is an abstract average value, around which the actually reported numbers of explained variance will fluctuate. When researchers report data statistics, they may throw wide nets capturing numbers from a range of empirical information describing their data and study design. This may create a situation where the *range* of reported numbers for explained variance could span all the way from zero to almost 100. However, many of these numbers will be statistical noise, caused by everything from measurement errors through common method variance to p-hacked reporting (Simonsohn et al., 2014).

Our proposed explanation for the observed flat rate of 42.8% is therefore that researchers will aim for questions that are semantically arguable but not obvious enough to be identified as tautological. Methodological conventions contribute to a pattern where this is the maximum rate of mutually explained variance. Empirically determined effects may contribute to bigger or smaller fluctuations in this number from study to study but these will cancel each other as noise in the average statistics.

To comply with the literature on construct validation (Anderson and Gerbing, 1991; Hinkin and Tracey, 1999; Colquitt et al., 2019), we will henceforth refer to the targeted constructs “focus constructs”, and use the term “orbiting constructs” for the other constructs that are part of the study but supposedly independent as evident through their own factor structures.

## Methods

The main method used here is the attempt to reconstruct the relationships between variables in the 1565 studies using semantic values only. Details will be described below, but the main approach is to collect texts that define the variables used in the study such as groups of survey items making up the measurement instruments. The texts making up these variables are subjected to a latent semantic analysis that produces a matrix of semantic relationships between the variables. These matrices are similar to correlation matrices and can be used as input to a principal component analysis (PCA). The ultimate objective is to produce a PCA that replicates the number of factors used in the study and check the extent to which the semantically identified structures will be able to explain each other.

As previously explained, the present exploration is only attempting to find if there does exist at least one possible semantic structure in our sample of studies. We think this suffices for theoretical reasons as there are empirical reasons why observed relationships may depart from the semantically expected. Also, it turns out that many publications do not offer sufficient original text materials to allow complete semantic reconstructions of all the data in the study. Reconstructing the semantic properties of studies requires a minimum amount of text to define variables and enter them into a semantic analysis. Working on variable level we would need texts such as the survey items making up measurement scales or variable definitions of some length. For most of the studies we work on item levels, but in a few rare examples the authors used single-item approaches and even only cited the variable definitions, in which case we could apply the same logic. We frequently find that the variable definitions or items used are taken from previous studies, which in turn themselves have used previously published variables. In this way, the original texts of definitions or items tend to recede backwards in time and sources, sometimes even behind paywalls or copyright protection.

Our approach has been to make a few attempts to find the original texts, but rather quickly abandon long searches and instead move downwards on a randomized list of our 1,565 articles. After 50 reasonably successful semantic reconstructions, we stopped the search as the statistics seemed to settle within confidence levels suggesting that further searches would not yield more information. The resulting set of studies originate from 13 different nations, covers 205 different psychological constructs and spans topics from clinical psychology through nursing and criminal detention to marketing and tourism.

Again, as in Study 1, this raises a question about the representativity and sample size. It is important here to understand that the sample size of 50 is taken to be representative of the 1565 original articles (and not of the total universe of psychological studies). The criteria for representativeness and statistical power are simpler in this case. We want a sample of the 1,565 articles that allows us to estimate the ratio of focus to orbiting factor loadings within a 95% confidence interval to support the proposition that 42.8% explained variance. To this purpose, 50 articles with 205 constructs should be statistically sufficient to accept the precision of the confidence intervals (cfr. polling practices, Samohyl, 2020).

For each of the studies, the reconstructed list of survey items (in a few instances simply the variable definition) are entered in a simple, easily accessible LSA algorithm, available to the public at the website lsa.colorado.edu. Here, we use the Touchstone Applied Sciences (TASA) semantic space requesting the algorithm to perform a “document-to-document” analysis. This algorithm may be regarded as a bit simple and crude, as there are other approaches that could be more fine-tuned towards our present purpose (Arnulf et al., 2018b). The approach was chosen for its public availability making the study easily replicable. Also, the numbers obtained from this service can be taken as conservative estimates of the degree to which semantics pervade the measurements.

Working in this way with LSA we could obtain matrices similar to a correlation matrix, with the difference that the LSA matrix merely contains numbers based on the texts with no knowledge about how humans might respond (Arnulf et al., 2018b). We then use this matrix as input to a principal component analysis (PCA) in [R], using the “principal” command in the Psych package (Revelle and Revelle, 2015) and asking for the number of factors used in the study, using varimax rotation.

In this procedure, we do not claim to replicate the exact statistics reported for each single study. That would require a meticulous attention to details that are hard to re-create and even harder to compare across the sampled studies. Instead, what we are aiming for is a two-step process that will go a long way in illuminating our research questions:

1) Does the LSA parsing of the measurement items demonstrate a detectable factor structure similar to the one reported in the original study? This implies in practice that the semantic values allow the PCA to yield satisfactory factor loadings with significantly lower cross-loadings. If so, the semantic structure inherent in the design must be assumed to influence the survey responses as shown in previous research.
2) We predicted that the ratio of cross loadings to all focus factor loadings will never exceed 50% and rather home in on around 40%. For all variables, regardless of their status as independent or dependent variables, we calculate how well they stand out from other variables based on semantics alone. If the average rate of cross-loading orbiting factors to focus factors is similar to what we observe, it will support our argument that the observed flat trend in explained variance is due to the semantic conditions adhered to in the prevalent designs of empirical psychological studies. In other words, the semantic relationships combined with methodological conventions set up the Bayesian priors that we expect to shape the observed values.

In the first step, to establish whether the factors allow replication through semantics, we look for the following criteria: We sum up the factor loadings of the items making up each construct/scale, and that are *unique* to that scale. The sum of factor loadings in the focus constructs are then compared to the factor loadings for the same items on the orbiting scales, and the difference in factor scores between focus and orbiting constructs is tested for statistical significance in a 2-tailed t-test. The t-test is done to ascertain that the semantically obtained factor does indeed stand significantly out from the orbiting factors. This procedure is done once for each single construct, then for the whole study (all factor loadings on focus constructs tested against all orbiting factor loadings). To replicate the factor structures through semantics, we require that the average factor loadings of focus constructs should be significantly higher than the average factor loadings of orbiting factors. From a methodological point of view, it is important to take into account that LSA is an approximation of how humans would perceive meaning in items, not an accurate prediction. Therefore, the accumulated statistics rather than each single construct or study will be of interest.

In the second step, we look at the ratio of orbiting factor loadings to focus factor loadings. The methodological literature contains various recommendations for how high factor loadings should be within a construct, and the maximum level of cross-loadings allowed. However, regardless of which convention one chooses to follow, the ratio of orbiting to focus factor loadings will give an estimate of how much these other factors may explain variation in the focus construct. Assume, for example, that factor loadings of a focus construct exceed 0.50, and the factor loadings of orbiting factors do not exceed 0.30. If, in a given study, the focus constructs have an average factor loading of 0.60 and the orbiting factor loadings have average loadings of 0.24, it implies that any of the orbiting factors will explain, on average,0.24/0.60 = 40% of the variance in the focus factors.

When these relationships are established based on semantic information alone, it simply means that the semantic structure in the study design is set up *a priori* for variables to explain each other at around 40%. It is important to bear in mind also that this is just an average number. Any individual pair of variables may show higher or lower values but our argumentation above presupposes this: High factor loadings indicate similarities indicating construct overlap, low factor loadings indicate different variables, but the ratio of orbiting to focus variables will indicate the degree to which we will expect the variables to explain each other. Empirical studies may show conditions where these relationships are skewed, but that would count as conditions in need of explanations.

## Results

The full list of included studies and the obtained statistics can be found in Table 3.

**TABLE 3:** Semantically replicated factor structures with fit statistics, topics and countries of origin.

Study no	Focus	Orbit	Orb/Foc	*P* value	Items	Item pairs	# Factors	RMSR	Chi2	Diag. fit	Topic	Country
1	0.40	0.09	24%	0.000	20	190	5	0.08	11,671.42	0.72	Personality	Spain
2	0.58	0.10	17%	0.000	24	276	5	0.06	10,372.11	0.96	Nursing	Taiwan
3	0.46	0.09	19%	0.000	38	703	7	0.05	18,727.26	0.94	Ethical leadership	Holland
4	0.74	0.29	40%	0.000	14	91	4	0.03	798.20	1,00	Technology acceptance	Germany
5	0.70	0.08	11%	0.000	20	190	5	0.05	5,703.58	0.97	Consumer psychology	Norway
6	0.73	0.35	48%	0.000	16	120	3	0.04	1,609.51	1,00	Physical education	Japan
7	0.57	0.10	17%	0.000	14	91	3	0.07	456.98	0.94	Medication therapy	USA
8	0.71	0.19	27%	0.000	13	78	3	0.06	2,806.15	0.98	Narcissism	USA
9	0.44	0.13	28%	0.000	19	171	4	0.08	12,016.31	0.83	Tourism	Thailand
10	0.58	0.39	67%	0.012	16	120	3	0.05	3,164.33	0.99	Pain treatment	Italy
11	0.75	0.13	18%	0.000	11	55	4	0.06	1,898.12	0.98	Consumer marketing	Germany
12	0.83	0.37	44%	0.000	9	36	3	0.01	4.51	1,00	Consumer loyalty	Norway
13	0.60	0.28	46%	0.000	22	231	3	0.08	13,398.91	0.97	Self-concept in classroom	Spain
14	0.40	0.35	88%	0.700	12	66	2	0.10	6,925.35	0.86	Nursing	Sweden
15	0.46	0.18	40%	0.000	23	253	5	0.06	8,695.35	0.98	Gambling cognition	Australia
16	0.65	0.08	12%	0.000	13	78	3	0.05	1,787.50	0.98	Autism and parenting	USA
17	0.71	0.35	49%	0.000	9	36	3	0.05	951.09	0.99	Work engagement	Holland
18	0.70	0.19	27%	0.000	13	78	3	0.05	4,421.20	0.98	Nurse-Physician relationship	USA
19	0.49	0.24	49%	0.000	24	276	5	0.05	7,545.36	0.99	Mindfulness and depression	Holland
20	0.65	0.13	20%	0.000	25	300	5	0.05	8,896.96	0.98	Resilience and anxiety	USA
21	0.58	0.23	39%	0.000	47	1081	4	0.05	2,611.62	0.99	Mindfulness and identity	UK
22	0.64	0.45	71%	0.002	18	153	2	0.06	5,903.56	0.99	Committed action	Sweden
23	0.46	0.24	52%	0.000	40	780	3	0.08	48,999.36	0.94	Psychosocial care	Sweden
24	0.63	0.22	35%	0.000	35	595	3	0.05	16,391.79	0.99	separation anxiety	USA
25	0.57	0.05	8%	0.000	57	1596	3	0.07	8,656.45	0.96	Children’s sleep	Israel
26	0.49	0.19	39%	0.000	20	190	3	0.06	7,931.30	0.96	Detention punitiveness	USA
27	0.56	0.53	95%	0.703	17	136	2	0.06	426.31	0.99	Management	USA
28	0.41	0.16	38%	0.000	24	276	4	0.06	11,562.92	0.90	Sex role identity	Germany
29	0.57	0.34	60%	0.001	15	105	3	0.06	3,686.10	0.99	Self perfectionism	USA
30	0.42	0.25	59%	0.015	26	325	3	0.09	25,534.58	0.93	Eating disturbances	USA
31	0.46	0.27	59%	0.000	26	325	6	0.03	2,419.06	1,00	Self-compassion	USA
32	0.55	0.21	38%	0.000	67	2211	3	0.07	10,0251.20	0.97	Eating disturbances	Germany
33	0.45	0.13	29%	0.000	49	1176	5	0.05	29,792.21	0.97	Occupational medicine	Norway
34	0.62	0.23	37%	0.003	9	36	3	0.10	392.45	0.92	Diabetes care	India
35	0.42	0.09	23%	0.000	28	378	7	0.06	1,408.55	0.90	Sex and gender identity	Brazil
36	0.56	0.10	19%	0.000	15	105	5	0.07	5,695.38	0.91	Crime sentencing	Canada
37	0.43	0.20	47%	0.009	15	105	4	0.07	5,423.48	0.94	Course management	USA
38	0.50	0.22	44%	0.005	13	78	5	0.03	693.22	1,00	Cancer treatment	Holland
39	0.48	0.31	66%	0.007	19	171	4	0.05	4,957.83	0.99	Behavioral activation	USA
40	0.51	0.29	57%	0.000	27	351	4	0.01	313.02	1,00	Impulsivity and risk	USA
41	0.40	0.16	41%	0.000	27	351	6	0.03	2807.57	1,00	Spiritual care	Holland
42	0.55	0.25	45%	0.000	23	253	3	0.04	4,534.88	0.99	Depression	Germany
43	0.50	0.35	70%	0.013	20	190	4	0.04	2,578.07	1,00	Financial worry	Holland
44	0.57	0.34	61%	0.024	9	36	4	0.04	516.54	1,00	Transport attitudes	Norway
45	0.48	0.10	22%	0.000	38	703	8	0.05	15,515.84	0.97	Professional practice env.	USA
46	0.48	0.19	39%	0.000	36	630	5	0.05	14,682.73	0.98	Constructive thinking	Holland
47	0.50	0.38	76%	0.059	22	231	3	0.05	5,784.24	0.99	Burnout inventory	USA
48	0.30	0.12	41%	0.000	56	1540	8	0.02	6,328.55	1,00	Burnout and thinking	Holland
49	0.62	0.24	38%	0.000	28	378	5	0.04	676.57	0.99	Math beliefs	USA
50	0.79	0.13	16%	0.000	17	136	4	0.05	3,214.98	0.99	Creativity at work	USA
Avg.	0.55	0.22	41%	0.031	24	361	4	0.05	11,864.03	0.96		

It appears that in all studies, the focus factors had higher factor loadings than the orbiting factors. In all studies but three (94%), the difference between the focus and the orbiting factor loadings were statistically significant. To describe the fit statistics of the model, we also display the standardized root mean square residual (SRMR) which should ideally be < = 0.05 (Shi et al., 2018). Moreover, since the LSA values are not based on variances in sampled scores, we only have the LSA matrix to work with and hence compute the diagonal fit as an indicator of model fit (Revelle and Revelle, 2015). Again, in all studies but three, the fit statistics also ranged from acceptable to very good and the average SRMR is 0.05. The mean difference between focus and orbit factor loadings is 0.34 (*p* < 0.001).

Looking at the numbers for the 205 constructs separately, there is as expected some more noise. For 184 out of 205 constructs (90%), the focus factor loadings are higher than the orbiting factor loadings. In 131 (64%) of these cases the differences were statistically significant. The average factor loading for focus constructs was 0.53 (stdev = 0.21), and the average for the orbiting factors was 0.20 (stdev = 0.12), with an average *p*-value for the difference of 0.15 (stdev = 0.25).

**FIGURE 3:**
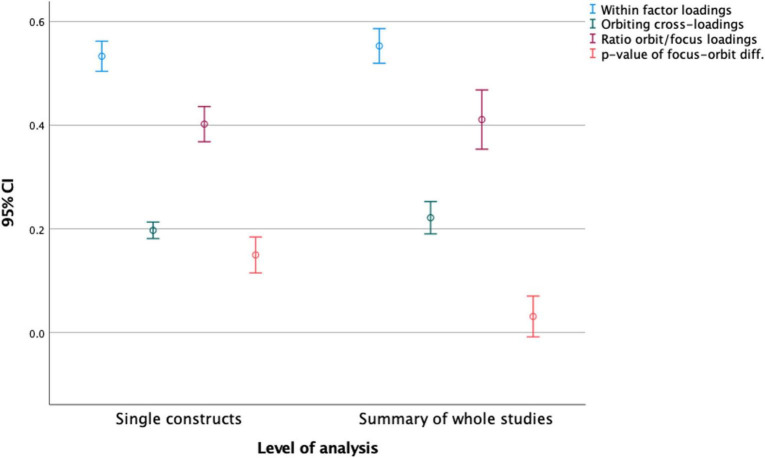
Means for focus and orbiting construct factor loadings, orbit to focus ratio and *P*-values.

This implies that the crude semantic algorithm used in this study was capable of detecting the semantic framework of factor structures in at least 64% of the individual constructs, a number raised to 94% on the level of single studies. Step one of our analysis was thereby successful. Moreover, the semantic structures replicated the established practice of taking high factor loadings as indication of common factors, while keeping cross-loadings low (Hinkin and Tracey, 1999). As shown in Figure 3, the confidence intervals of the obtained values indicate that there should be very little variation in the existing population of such studies and hence, we think it is warranted to stop our search for more articles to reconstruct.

Addressing step two in our analysis, we explore the confidence interval for the ratio of orbiting to focus factor loadings as presented in Figure 3. This ratio is 0.22/0.55, which equals 40%. Taken literally, this means that the average explained variance between any constructs in these studies will be 40%, based on semantics alone. As indicated in Figure 3, the 95% confidence interval spans from the high 30 s to the low 40 s. These numbers differ slightly depending on whether full studies (*N* = 50, Orbit/Focus ratio 40%) or single constructs (*N* = 205, Orbit/Focus ratio = 37%) are used in the calculations.

What this implies, is that the conventions of creating factor structures on data that are semantically determined will usually bring together variables that may explain more than a third of each other. The reason why our dataset contains explained variances at the higher end of this estimate (42.8%) is probably due to two main factors: One, because the researchers will most likely look for relationships that can be semantically (logically) argued, and second, because of a bias toward reporting salient findings. The semantic structure of study designs in psychology creates conditions for *a priori* correlations, but to comply with rules for factor structures, internal consistency and maximum levels of cross-loadings, the explained variance will probably rarely exceed 45% without appearing as a blatant tautology.

The average factor loadings for the focus variables are significantly different from 0.50, such that the likelihood of other variables explaining more than 50% is a rare incident. This applies both at individual construct level and at aggregated study levels. Conversely, the orbiting factor loadings are always significantly different from 0, meaning that the semantically pre-determined correlations will always be present to influence or even determine the shared variance between measures in a study. The ratios of orbiting to focus factor loadings, however, are not significantly different from 43%. While the average ratio is lower, there are enough cases left where researchers can happily claim to have found a large effect ranging around 40%.

**FIGURE 4:**
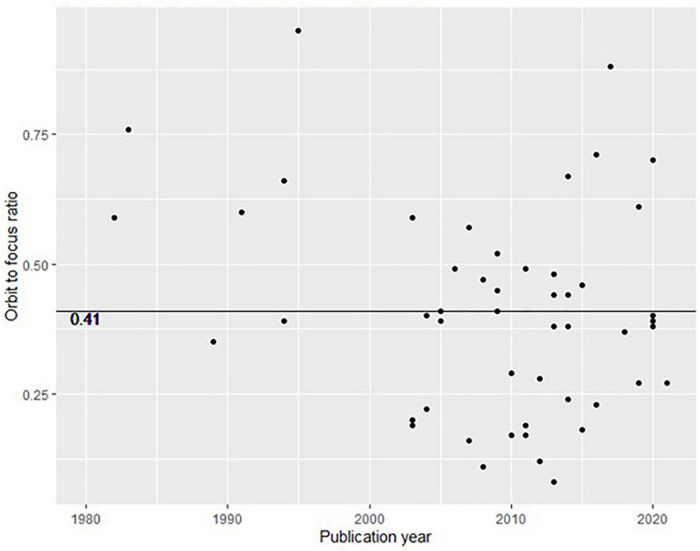
Plotted ratios of orbiting to focus factors (similar to explained variance in Figure 2).

We plotted the orbit-to-focus ratio in Figure 4, such that the semantically explained variance between all constructs are displayed. The emerging picture in Figure 4 is structurally similar to the one displayed in Figure 2, although Figure 4 is based on the semantic reconstruction of only 50 publications sampled from the 1565 publications plotted in Figure 2.

Moreover, we listed all the 205 included single constructs in Table 4. Analogous to Table 2, it shows that a broad range of psychological topics are included in our sample and does not stem from a particular field or research community.

**TABLE 4:** Constructs included in the semantic factor analyses.

Construct (factor) names				
Personal support and patient counseling	Maternal separation anxiety	Emotional exhaustion	Manager support	Dedication
Acceptance of students with disabilities	Chronic offending lifestyle	Affective commitment	TAM ease of use	Absorption
Financial rumination-related cognitions	General health satisfaction	Emotional investment	Service quality	Dedication
Behavioral activation reward response	Inclusion versus exclusion	Cultural sensitivity	Common humanity	Detachment
Deterrence	Inability to stop gambling	Over-identification	Eating disorder	Leadership
**Attitude toward patient spirituality**	**Concern for sustainability**	**eCollege preference**	**Self-evaluation**	**Resilience**
Financial rumination-related emotions	Creative personal identity	Illusion of control	Risk perception	Self-blame
Professionalization of spiritual care	Physical exercise regimen	Generalized anxiety	Female-positive	Efficiency
Handling disagreement and conflict	Medication administration	Delivery preference	Female-negative	Narcissism
Staff relationships with physicians	Committed action positive	Higher living costs	No future value	Compliance
Socially prescribed perfectionism	Committed action negative	Role clarification	Intent to stay	Commitment
Learning and following from others	Referral to professionals	Self-perfectionism	TAM usefulness	Aggression
Financial worry-related cognitions	General life satisfaction	Gender orientation	Self-judgment	Integrity
Behavioral activation fun seeking	Math confidence dimension	Sexual orientation	Communication	Isolation
Management	Perceived training needs	Classroom behavior	Personal norms	Procedure
Perceived relative attractiveness	Positive urgency measure	Predictive control	Habit strength	Act-aware
Implementation of spiritual care	Relationship exclusivity	Follow-up icu care	Economic costs	Non-judge
Rehabilitative motivation index	Internal work motivation	People orientation	Self-kindness	Non-react
Amount of organizational support	Erotophilic disposition	Discouraging words	Safety system	E-loyalty
Financial worry-related emotions	Personal accomplishment	Creativity at work	Work pressure	Enjoyment
Cross-application of experiences	Sandbagging	Willingness to pay	Special needs	Activity
Leadership in clinical practice	TAM behavior intention	Sensation seeking	Participation	Male-all
Punishment/control orientation	Calculative commitment	Job stress index	Core deficits	Fairness
Positive other-oriented style	Supports local economy	Sexual constraint	Religiousness	Capacity
Positive self-oriented style	Sexual attractiveness	Procedural rights	Power sharing	Describe
Medication therapy management	Creative self-efficacy	Interpretive bias	Male-positive	Strength
Other-oriented perfectionism	Customer satisfaction	Mental well-being	Male-negative	Workload
Co-morbid behavioral symptoms	Personal interactions	Physical problems	Effectiveness	Fairness
Negative self-oriented style	Dichotomous thinking	Physical activity	Extraversion	Attitude
Positive self-oriented style	PowerPoint preference	Magical thinking	Peer support	Behavior
Negative self-oriented style	Emotional exhaustion	Depersonalization	Oral control	Teamwork
Positive self-oriented style	Gambling expectancies	Intended loyalty	Unit support	Dieting
Behavioral inhibition system	Social identification	Child temperament	Satisfaction	Bulimia
Self-oriented perfectionism	Psychosocial problems	Economic benefits	Active trust	Observe
Extent of personalizing care	Maternity separation 1	Class devaluation	Goal setting	Meaning
Co-morbid physical symptoms.	Maternity separation 2	Depersonalization	Infant sleep	Comfort
Communication about patients	Maternity separation 3	Goal achievement	Math anxiety	Mystery
Behavioral activation drive	Environmental concern	Ethical guidance	Neuroticism	Coping
Behavioral activation drive	Control over practice	Female-all	TAM quality	Vigor
Mindfulness	Self-esteem		Competence	Time

In section 3 we attempt to explain the results of section 1 & 2 and to provide a possible explanation for why psychology has not increased its ability to explain variance and why semantic algorithms produce almost identical results as studies that collected data.

## General discussion

“What is new in psychology is not good and what is good is not new”^1^

What can one learn from the findings reported here? One way to answer this is to ask how one could explain any alternative outcome.

What if we had found an *increase* in universal predictability over the years? This would have been in accordance with the common assumption that psychology is “advancing,” but it would also have meant that empirical research must have discovered some previously unknown universal regularities or laws. One can only predict what is repeating. Considering the great variety of studies and multitude of constructs involved, we may infer that psychology has not been able to come up with new universal laws. Since one cannot formulate or conceive of a possible such law, it would appear that universal predictability (42.8%) must be taken to be constant and interpreted as *non-contingent a priori* (Bradley and Swartz, 1979), p. 157) given the human communicative system.

What if we had found that what *is* universally predictable, could *not* be entirely derived from semantics. This would have meant that research must have had discovered some previously unknown universal laws, *not derivable from semantics.* Considering the great variety of studies and constructs, it appears to be impossible to conceive of and formulate such laws. Everything universally predictable in psychology seems to derive from semantics.

Summarizing the preceding, it appears that asking “what if” our results had come out differently, leads to exceedingly implausible alternative answers.

At this point, we will mention some historical predecessors and then try to formulate a unitary explanation of our findings.

The modern history of psychology can be said to have begun with attempts to imitate the methods and successes of the natural sciences. From the very beginning, this project exposed a deep conflict between what Dilthey (Rickman, 1976) labeled “explaining” and “understanding” psychology. The former approach focuses on the causes, and the latter focuses on the meanings of psychological phenomena. This conflict between natural science and hermeneutic approaches has persisted until today, but the causal project has dominated at the universities and the hermeneutic in psychological practice. The transition from “arm-chair psychology” to experiments, has been described by Danziger (1990) and many others.

Here, we want to point to the early transition from studying individuals to studying group averages. After 1900 almost all publications reported average data from many subjects, and academic psychology became the study of the “generalized human mind.” The background for this unobtrusive early change has become very directly visible after the publication of the “Urmanuskript” by Ebbinghaus (1880/1983), Smedslund (1985). This was Ebbinghaus’s hand-written “master’s thesis” and was the first and only serious published effort to do a natural-science- type experiment (varying one factor and keeping all others constant) on the psychological processes in *one* person. He attempted to study the effect of chronological time on amount of remembered material. For the present purpose it should suffice to mention that, even with the ingeniously designed material of lists of “non-sense syllables,” he reported serious and persistent difficulties in keeping constant indefinitely numerous and complex variables (including factors such as time since last meal). Only by means of statistical analyses of very extensive data did he arrive at a “law of forgetting” consistent with the age-old common-sense rule that “as time goes by, we tend to forget more”.

*No one* attempted to repeat Ebbinghaus’s study, but the exact reasons why everyone began to use average results of many individuals were not much debated. Over the following years, empirical research in psychology has nevertheless led to increased recognition of the serious difficulty of controlling the indefinitely complex and ever-changing *context*, vividly described by Ebbinghaus (Barrett et al., 2021).

The continuing methodological difficulties of keeping conditions constant were accompanied by a diminished interest in looking for context-independent universal laws (Teigen, 2002). Roediger (2008) concluded that psychological regularities always are *relative.* Psychological phenomena are determined by indefinitely complex contexts, far transcending the here-and-now, whereas natural science data are determined by a context of a limited number of exclusively here-and-now factors and therefore allow universal laws.

**FIGURE 5:**
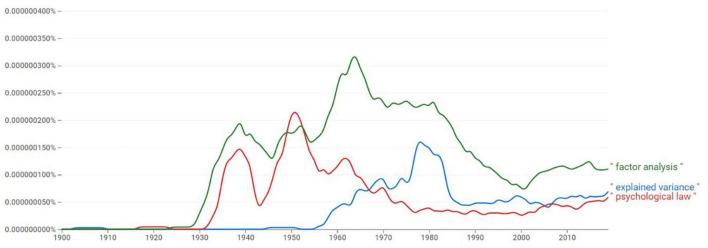
The relative prevalence of the terms “psychological law,” “factor analysis,” and “explained variance” in the English language throughout the years 1900-2010 (Source: Google nGram viewer).

The historical movement away from pursuing “psychological laws” toward citing effect sizes like explained variance is visible in Figure 5. The prevalence of concepts in the English language as mapped by Google suggests a decline in the usage of “law”, concomitantly with an increase in “factor analysis” that, in turn, coincides with a spark in usage of “explained variance.”

The causal approach to psychology changed not only from individuals to group averages but, thereby also from direct cause-effect studies to the much weaker criterion of statistical significance. This, in turn, allowed a widespread fallacy of interpreting statistically significant results as tests of given hypotheses, ignoring possible alternatives and thereby leading to a senseless accumulation of allegedly “supported” hypotheses (Green, 2021). In addition, (Lamiell, 2019) showed that the *psycho-demographic* research (based on group-averages) yields “no information whatsoever about processes in individuals.” This conclusion raises questions about the alleged usefulness of academic research for practice.

In summary, the difficulty of keeping conditions constant given the indefinitely complex ever-changing contexts, forced the project of a causal, natural-science type psychology to change into psycho-demography. In addition, consistent misinterpretation of tests of statistical significance led to a deteriorating research tradition producing endless numbers of “confirming” findings, and no theoretical advance (Haeffel, 2022). For the mentioned reasons, the prevailing “psycho-demography” approach cannot be expected to result in generally improved prediction (discovery of universally valid new theories), which accords with the present findings.

The other main approach, *“the understanding psycholog*y” (Palmer, 1969) continues the earlier hermeneutic traditions and has led to numerous theories about processes in individuals and attempts to predict, but there is no common methodology allowing for empirical estimation of progress. However, there has been definite theoretical advancement allowing us to explain our second main finding, namely that what *can* be predicted is wholly derivable from semantic algorithms.

To *understand* what an experience or act x *means* is to know what *follows from* x and what x *follows from* (Smedslund, 1970). This includes classical logical tautologies, that is, what is implied by the definitions of the terms. Example: If a person experiences something unexpected, she will be surprised. If “surprise” is defined as “the state of having experienced something unexpected.” this proposition is necessarily true. Logical necessity is also found in the more complex example given by Heider (1958). “The statement that somebody who can do something and tries to do it will succeed in doing it, is analytic, and does not have to be proven by experiment” (p. 297). However, Heider’s example raised the general question of how propositions can be proved in the absence of strict definitions of the ordinary language words. Smedslund (1988) tried to develop a formal axiomatic system by supplying such definitions. However, this turned out to be difficult given the fuzziness of ordinary words and therefore, the system was changed by replacing definitions with theorems (Smedslund, 1997). For example, terms such as “frustration” and “anger” were no longer formally defined, but the theorem “if P is angry, then P is frustrated” was retained. In this way, logical relations in the otherwise fuzzy system of ordinary language were preserved.

The assumption of a fixed axiomatic structure of language also accords with the thinking of Lakatos (Lakatos, 1978) who points to a hard core of theoretical assumptions that cannot be abandoned or altered. In the same vein, Wierzbicka (Wierzbicka, 1996) argues for “the existence not only of an innate and universal “lexicon of human thoughts” (the 62 + primitive concepts), but also of an innate and universal “syntax of human thoughts.” The primitive concepts cannot be defined, but together form a system (the Natural Semantic Metalanguage) that is common to all the thousands of human languages.

The primitive concepts are not independent but form a system with considerable logical constraints. For example, the five mental concepts KNOW, THINK, WANT, FEEL, and DO all influence each other (what I KNOW influences what I THINK, what I WANT, what I FEEL, and what I DO, etc.) See also Dennett (Dennett, 1987).

Semantic theory points to two general characteristics of words, namely polysemy (the same word can have different meanings) and allolexy (the same meaning can be expressed by different words). The extent of the polysemy (how many meanings a word can have) and the extent of the allolexy (how many ways a meaning can be expressed) is generally taken to be indefinite.

The results reported in the present article, accord with a converging conclusion reached by both the causal and the hermeneutic approaches to psychology.

It appears that both approaches to psychology are recognizing the commonsense truth that *a given human act can be caused by indefinitely many circumstances and can* mean *indefinitely many things.* This statement can be shown to be true by formulating its negation: “A given human act is caused by only one circumstance and can mean only one thing” or “A given human act is caused by a definite number of circumstances and has a definite number of possible meanings” (van Holthoon and Olson, 1987; Siegfried, 1994).

It remains to formulate this converging insight in a unified terminology and show how it accords with the findings reported here. We think this can be done by means of the notion of *context.*

Context is an age-old concept, and a necessary part of every complete psychological description. Here, we introduce an interpretation of the word that can be used in both the causal and the hermeneutical approaches and which, in terms of both paradigms has definite consequences for psychological theory and practice.

Context is determining (causing/implying) psychological phenomena and processes. Here, we take our departure in the following delimitation of the concept:

*The context of a person’s experience and acting is everything that, if changed, changes this experiencing and acting.*

We also assume that:

*Psychological processes are determined by indefinitely complex and irreversibly changing contexts.*

This proposition cannot be proved or refuted but states what we think must be taken for granted.

“Indefinitely complex” means that it is impossible to ascertain that one has exhaustibly described a psychological context. One cannot eliminate this uncertainty, because in everyday life, as well as in experiments, one can always envisage additional or alternative possibilities.

“Irreversibly changing” means that there is no opposite process (one cannot “un-experience,” “un-act,” “un-develop.”) Therefore, given the indefinitely complex and irreversibly changing contexts, replication is never possible.

The lack of advance in what can be predicted follows directly from the assumption that t*he context of psychological processes is indefinitely complex and irreversibly changing.* Prediction is only possible to the extent that there is invariance or regularity, and an increase in predictability must mean discovery of hitherto unknown universal laws.

In order to understand why this is so, we consider the outcome of a study by Smedslund (1970) in which subjects failed to learn to utilize information from three probabilistic cues, with a multiple correlation of + 0.93 with a numerical criterion. Up to 4,800 trials there was no evidence of learning, that is no increase in predictability. The finding was explained in terms of the biological principle that there can be no *accommodation* without *assimilation* (Piaget, 1950). In psychology, this can be reformulated *pace* Wittgenstein, as “one cannot learn a relationship (discover a regularity) unless one can put words on the invariantly related entities.” The failure to learn in the study by Smedslund can perhaps be seen as a partial miniature analog of the failure to advance predictability in psychology as a whole. In both cases one may attribute the lack of advance to our inherent cognitive limitations or the indefinitely complex context. Anyhow, one must conclude that, in terms of predictability, the project of modern psychology has failed.

The considerable amount of predictability reported here, and that presumably has always existed, can be explained by the rule-regulated social life. Humans cope with the indefinitely complex, irreversibly changing contexts by *communicating* and *cooperating*, that is, by means of language. This allows them to increase predictability by relying on rules, that is, on what is stipulated, agreed or promised. Imagine, for example, two persons who agree to meet in a hotel in Kuala Lumpur at a given date. Despite the indefinitely complex possibly adverse circumstances, the meeting is likely to take place. Social life reduces the effects of the indefinitely complex and irreversibly changing context. To allow human communication and linguistic competences, the human language must itself give rise to a relatively stable, inter-subjective system of meaning. A fact that most people take for granted, but is remarkable from a cognitive perspective, is the relative speed and precision with which we process linguistic structures (Michell, 1994; Poeppel et al., 2012). Linguistically, humans are “competent without comprehension” (Dennett, 2009), and thus we do not seem to differ sufficiently between the structures of language and the structures of the world – the map and the terrain (Arnulf, 2020). The constant predictability (42.8%) reported here can potentially be explained as the total effect of the allegedly universal genetically determined, human communicational system. The 60 + primitive concepts and syntax proposed by Goddard and Wierzbicka (2002) form an attempt to describe such a system and any possible improvements in psychological theories must take this representational nature of language into account.

## Limitations and further research

The propositions and empirical explorations in this study do of course have limitations. We have taken the liberty to adopt one possible criterion for “progress”, i.e., explained statistical variance, and follow up on this single measure. It is definitely possible to judge progress using other criteria and one motivation for the present article is indeed to welcome such a discussion. In line with our somewhat myopic perspective, it is also possible to question the criteria for representativity applied here.

Again, it is these criteria that we want to address. One of the authors in this study has a personal history of more than 70 years of scientific publications in the field of psychology. From his point of view, the discipline has evolved from a search for universal laws with predictive powers to a reliance on statistical support for smaller sets of variables, a tendency labeled by some scholars as “statisticism” (Lamiell, 2013, 2019). There seems to be a real danger that this tendency leads to a reduction in the scientific impact of research findings in social science (Elster, 2009, 2011, 2018).

It is therefore possible that there is psychological research being published that does not meet the criteria we have applied in the present study. For example, recent advances in cross-cultural psychology have called attention to the fact that many psychological constructs are cultural variables rather than fundamental constants, inciting efforts to re-conceptualize the way we theorize and carry out empirical tests (Henrich et al., 2010). We do however think that the sampling and statistical procedures we apply here are representative of the mainstream reporting of effect sizes and explained variances. We call for and welcome attempts to counter our findings, as we believe this discussion would vitalize the conceptual foundations of psychological methodology.

## Conclusion

This paper started with an attempt at measuring the progress of scientific psychology by means of explained variance in PsycINFO. The main finding is probably highly surprising to most readers: that explained variance has been constant in the period from 1956 to 2021. In the second part, we found that the results from the first section could be replicated based on a semantic analysis, that is, without collecting any data. We have tried to explain why the findings could not have turned out differently. It seems very difficult to conceive of and explain a hypothetical universal increase in predictability, or that the existing predictability would not be derivable from language. This leaves us facing serious questions and implications both for research and practice.

## Data availability statement

Publicly available datasets were analyzed in this study. This data can be found here: https://www.apa.org/pubs/databases/psycinfo.

## Author contributions

GS contributed the original idea and provided data and analyses on explained variance. JA extracted the subgroup of studies and performed LSA- and factor analyses. JS drafted the general discussion. All authors contributed to writing the manuscript.

## Footnotes

1 Quoted by David Krech, in a lecture in 1949, while Krech was a visiting professor at the University of Oslo, Norway.
